# Hypoxia-induced one-carbon metabolic reprogramming in glioma stem-like cells

**DOI:** 10.1093/lifemedi/lnad048

**Published:** 2023-11-23

**Authors:** Xuan-Cheng He, Jian Wang, Min-Yang Shi, Chang-Mei Liu, Zhao-Qian Teng

**Affiliations:** State Key Laboratory of Stem Cell and Reproductive Biology, Institute of Zoology, Chinese Academy of Sciences, Beijing 100101, China; Institute for Stem Cell and Regeneration, Chinese Academy of Sciences, Beijing 100101, China; Beijing Institute for Stem Cell and Regenerative Medicine, Beijing 100101, China; State Key Laboratory of Stem Cell and Reproductive Biology, Institute of Zoology, Chinese Academy of Sciences, Beijing 100101, China; Savaid Medical School, University of Chinese Academy of Sciences, Beijing 100049, China; State Key Laboratory of Stem Cell and Reproductive Biology, Institute of Zoology, Chinese Academy of Sciences, Beijing 100101, China; Savaid Medical School, University of Chinese Academy of Sciences, Beijing 100049, China; State Key Laboratory of Stem Cell and Reproductive Biology, Institute of Zoology, Chinese Academy of Sciences, Beijing 100101, China; Institute for Stem Cell and Regeneration, Chinese Academy of Sciences, Beijing 100101, China; Beijing Institute for Stem Cell and Regenerative Medicine, Beijing 100101, China; Savaid Medical School, University of Chinese Academy of Sciences, Beijing 100049, China; State Key Laboratory of Stem Cell and Reproductive Biology, Institute of Zoology, Chinese Academy of Sciences, Beijing 100101, China; Institute for Stem Cell and Regeneration, Chinese Academy of Sciences, Beijing 100101, China; Beijing Institute for Stem Cell and Regenerative Medicine, Beijing 100101, China; Savaid Medical School, University of Chinese Academy of Sciences, Beijing 100049, China

**Keywords:** glioblastoma, hypoxia, DHFR, MAT2A, tumorigenicity

## Abstract

Glioma stem cells (GSCs) in the hypoxic niches contribute to tumor initiation, progression, and recurrence in glioblastoma (GBM). Metabolic pathways are altered in GSCs under hypoxia, but the mechanism underlying the altered one-carbon metabolism in GSCs by hypoxia is largely unknown. Here, we report that hypoxia induces down-regulation of DHFR as well as up-regulation of MAT2A in GBM tumorsphere cells, and confers them the ability of cell proliferation that is independent of exogenous folate. Importantly, short-term inhibition of the methionine cycle or exposure to the MAT2A inhibitor is sufficient to cripple the tumor-initiating capability of GBM tumorsphere cells. Therefore, we present a novel perspective on how hypoxia alters the pattern of one-carbon metabolism in GBM tumorsphere cells and provide evidence that restriction of methionine intake or targeting MAT2A inhibits the tumorigenicity of GBM tumorsphere cells.

## Introduction

Glioblastoma (GBM) is one of the most malignant tumors, which grows quickly and still has no cure [1], highlighting an urgent need for understanding of this deadliest cancer and developing effective therapeutics. GBM displays complex cellular heterogeneity containing glioma stem cells (GSCs) that are responsible for disease progression, cancer invasion, tumor angiogenesis, therapeutic resistance, immune evasion, and tumor recurrence [2, 3], indicating that targeting GSCs may be crucial for preventing GBM recurrence.

Hypoxia is a well-known regulatory factor for the regulation of angiogenesis and stem cell biology [4–10]. Hypoxia promotes tumor progression by inducing neovascularization, elevating pro-tumor inflammation, enhancing therapeutic resistance, altering metabolism, and reinforcing the maintenance of cancer stem cells [11–14]. In the case of hypoxia, the expression of HIF-2α (but not HIF-1α) mRNA is up-regulated only in GSCs, but not non-stem cancer cells or fetal human neural progenitor cells [11]. HIF-2α-regulated genes, including *Oct4*, *Glut1*, and *SerpinB9*, are expressed at significantly higher levels in GSCs as compared to matched non-stem cancer cells under hypoxia [11]. Hypoxia-inducible factors (HIF) knockdown impairs tumorsphere formation not only in primary assays but also in secondary and tertiary passages, indicating the HIFs are required for proliferation of GSCs. Thus, hypoxia is a critical component of a cancer stem cell niche [9]. Recent studies have shown that USP33 deubiquitinates and stabilizes HIF-2α to promote hypoxia response in GSCs [15]. Hypoxia-induced GLT8D1 promotes the maintenance of GSCs by inhibiting CD133 degradation through N-linked glycosylation [16]. However, the mechanism underlying hypoxia-induced maintenance of GSCs remains to be fully explored.

One-carbon metabolism encompasses a series of interlinked metabolic pathways [17, 18]. One-carbon metabolism has various nutritional inputs including glucose, serine, threonine, methionine, glycine, choline, folate, and vitamin B12. One-carbon units from a major methyl donor, dietary folate, are transferred via a series of reactions to various methyl acceptors and recycled via by-product homocysteine (Hcy) by two cycles termed the methionine and folate cycles [19]. Altered one-carbon metabolism is critical for the rapid proliferation of cancer cells. Folate cycle enzymes, especially MTHFD2, a folate cycle enzyme, is overexpressed in various cancer cells and enable cancer cells to become more tolerant to effector T cells [20]. The methionine synthase MTR converts homocysteine to methionine and plays a dominant role in coupling folate and methionine metabolic cycles in the context of tumor. In the absence of folic acid, MTR incorporates 5-methyl tetrahydrofolate (5-mTHF) into the folate cycle to sustain intracellular folate metabolism [21]. Importantly, pharmacological inhibition of the methionine cycle or methionine adenosyltransferase II alpha (MAT2A) reduces protein synthesis rate and tumor cell growth [22] [23]. However, at present, the relationship between hypoxia and one-carbon metabolism in GSCs is still largely unknown.

Here, we reported that hypoxia causes down-regulation of dihydrofolate reductase (DHFR) and up-regulation of MAT2A in GBM tumorsphere cells, which results in reduced demand for folate but higher dependency on methionine cycle activity by GSCs. We demonstrated that limiting methionine intake or targeting MAT2A efficiently suppresses the tumorigenicity of GBM tumorsphere cells, thus present a novel perspective on hypoxia-mediated maintenance of tumor-initiating cells.

## Results

### Exogenous folate is essential for adherent tumor cells, but not for tumorsphere cells

**Figure 1.**
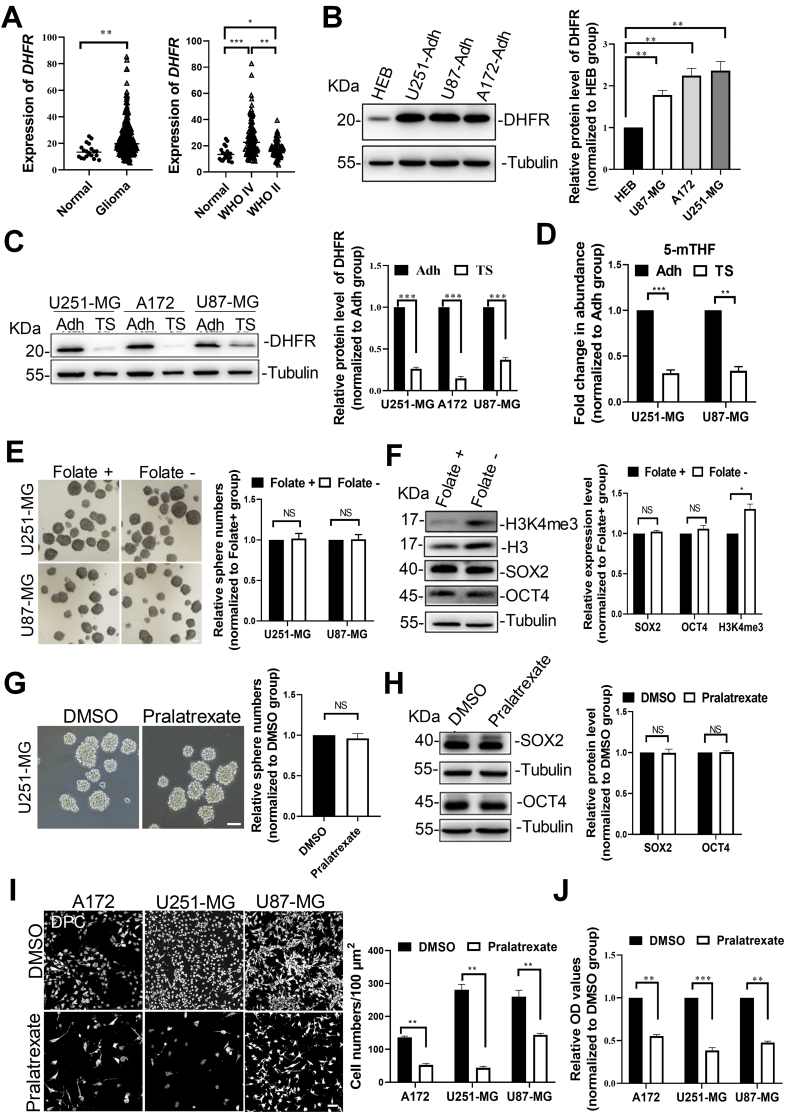
**GSCs but not adherent tumor cells did not require exogenous folate intake.** (A) Expression levels of *DHFR* mRNA in glioma samples from CGGA database. Normal brain tissue (*n* = 20), WHO grade II glioma (*n* = 71), WHO grade IV glioma (*n* = 103). (B) Protein levels of DHFR in normal brain glial cell line (HEB) and adherent cells (Adh) that were derived from three glioblastoma lines (U251-MG, U87-MG, and A172). Tubulin was used as a loading control in western blots. (C) Western blots showing protein levels of DHFR in tumorspheres (TS) and adherent tumor cells. Tubulin was used as a loading control. (D) Abundance of intracellular 5-mTHF in tumorspheres as determined by enzyme-linked immunosorbent assay (ELISA), normalized to abundance in adherent tumor cells. *n* = 3 biologically independent experiments. (E) Tumor-sphere formation assays were performed in medium with complete formulation or without folate supplementation for 10 days. White bar, 100 μm. (F) Protein levels of SOX2, OCT4 and H3K4me3 in U251-MG cells that were cultured in medium with complete formulation or without folate supplementation for 10 days. Tubulin and total H3 were used as loading controls. (G) Tumor-sphere formation assays were performed on U251-MG cells exposed to 10 μM of pralatrexate or DMSO for 10 days. White bar, 100 μm. (H) Protein levels of SOX2 and OCT4 in U251-MG tumorspheres exposed to pralatrexate or DMSO for 10 days. Tubulin was used as a loading control. (I) Representative images and quantifications of adherent tumor cells exposed to pralatrexate or DMSO for 2 days. DPC: digital phase contrast. White bar, 10 μm. (J) CCK-8 assay showing relative OD values of on adherent tumor cells exposed to 10 μM of pralatrexate or DMSO for 2 days. Data are presented as mean ± standard error of the mean (SEM). *P < 0.05, **P < 0.01, ***P < 0.001, NS: no significance.

Although folate supplementation is closely related with cell proliferation and one-carbon metabolism, its function in promoting or suppressing cancer development is highly controversial [24]. To examine whether folate affects the development of glioma, we analyzed the RNA-seq data from the CGGA database and found that higher mRNA levels of DHFR, a key player in folate metabolism, were significantly correlated with World Health Organization (WHO) grade II/IV glioma (Fig. 1A). Then, we compared the protein levels of DHFR in normal brain glial cell line (HEB), adherent tumor cells and tumorspheres that were derived from three glioblastoma cell lines (U251-MG, U87-MG, A172). Interestingly, DHFR was up-regulated in adherent tumor cells compared to HEB cells (Fig. 1B), but down-regulated in tumorspheres compared with adherent tumor cells (Fig. 1C). In addition, we found a dramatic reduction of 5-mTHF in tumorspheres, an evidence of decreased uptake of folate by tumorspheres (Fig. 1D). These results suggest that folate metabolism is altered in tumorsphere cells.

Next, we evaluated the effect of folate deficiency on tumorsphere formation by culturing tumorsphere cells in folate-deprivation medium. Quantitative analysis showed that folate deficiency did not alter the sphere-forming capacity (Fig. 1E) and the expression levels of pluripotency marker SOX2 and OCT4, but marginally up-regulated the expression of H3K4me3 (Fig. 1F). This indicates that tumorsphere cells do not require exogenous folate intake. To further confirm our findings, we examined any effect of pralatrexate, an inhibitor of DHFR, on the tumorsphere formation. Again, we did not observe any differences in the number of tumorsphere colonies (Fig. 1G) and in their expression of OCT4 and SOX2 (Fig. 1H) between pralatrexate and DMSO treatments. However, the number of viable adherent cells was severely reduced after administration of pralatrexate for 48 h as compared to mock treatment with DMSO (Fig. 1I and 1J). Thus, exogenous folate is required for survival and proliferation of GBM adherent tumor cells but not for tumorsphere cells.

### Methionine is an indispensable metabolic substrate for tumorsphere cells.

**Figure 2.**
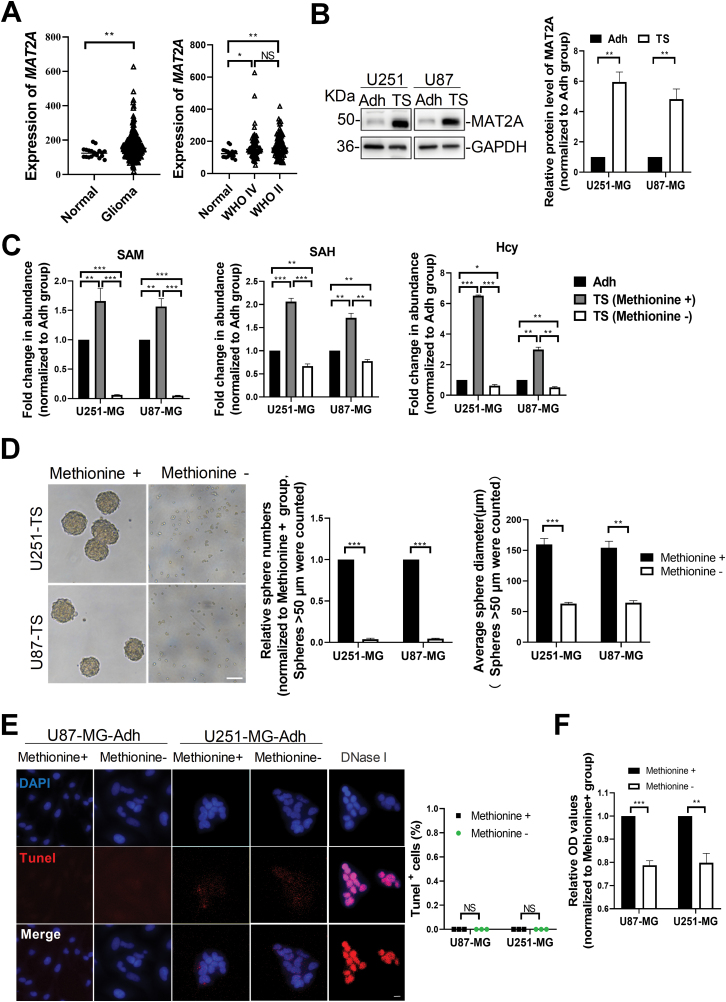
**Methionine is an indispensable metabolic substrate for GSCs.** (A) *MAT2A* mRNA expression levels in glioma samples from CGGA database. Normal brain tissue (*n* = 20), WHO grade II glioma (*n* = 97), WHO grade IV glioma (*n *= 78). (B) Protein levels of MAT2A in tumorspheres and adherent cells that were derived from U87-MG and U251-MG cell lines. GAPDH was used as a loading control. (C) Abundance of intracellular SAM, SAH, and Hcy in tumorspheres cultured with complete formulation or without methionine supplementation for 10 days as determined by ELISA, normalized to adherent cells group. *n* = 3 biologically independent experiments. (D) Representative images and quantification of tumorspheres derived from U87-MG and U251-MG cells which were cultured under complete formulation and methionine-null medium conditions for 10 days. (E) Representative images and quantifications of Tunel stainings on adherent tumor cells that were cultured in medium with complete formulation or without methionine for 2 days. DNase I was used as a positive control. White bars, 10 μm. (F) Relative OD values of adherent tumor cells after methionine starvation for 48 h, measured by CCK-8 assay. Data are presented as mean ± SEM. *P < 0.05, **P < 0.01, ***P < 0.001, NS: no significance.

The methionine cycle is another important unit of the one-carbon metabolism. By analyzing the RNA-seq data from the CGGA database, we found a higher level of *MAT2A* mRNA in WHO grade II/IV glioma than that in healthy brain (Fig. 2A). Interestingly, MAT2A expression was significantly up-regulated in tumorspheres compared to adherent cells derived from U251-MG or U87-MG (Fig. 2B). Consistently, we observed a dramatic increase of metabolites (S-adenosylmethionine [SAM], S-adenosyl-homocysteine [SAH], and Hcy) in tumorspheres compared to adherent cells derived from U251-MG or U87-MG cells which were cultured in medium with complete formulation for 10 days (Fig. 2C). These results suggested that the methionine cycle is more activated in tumorsphere cells than that in adherent tumor cells.

We then tested whether methionine is an indispensable metabolic substrate for tumorsphere cells. By culturing U251-MG or U87-MG cells in methionine-null medium for 10 days, the absence of methionine severely reduced both the number and the average diameter of tumorspheres (Fig. 2D). Since methionine serves as an essential amino acid, we examined the effects of short-term methionine starvation on cell survival and found that adherent tumor cells did not exhibit significant apoptosis after methionine starvation for 48 h (Fig. 2E). However, cell counting kit-8 (CCK-8) assay demonstrated that short-term methionine starvation did inhibit the proliferation of adherent tumor cells (Fig. 2F). These results suggest that methionine is required for the growth but not for the survival of tumor cells.

### Hypoxia is a key factor in altering the pattern of one-carbon metabolism

**Figure 3.**
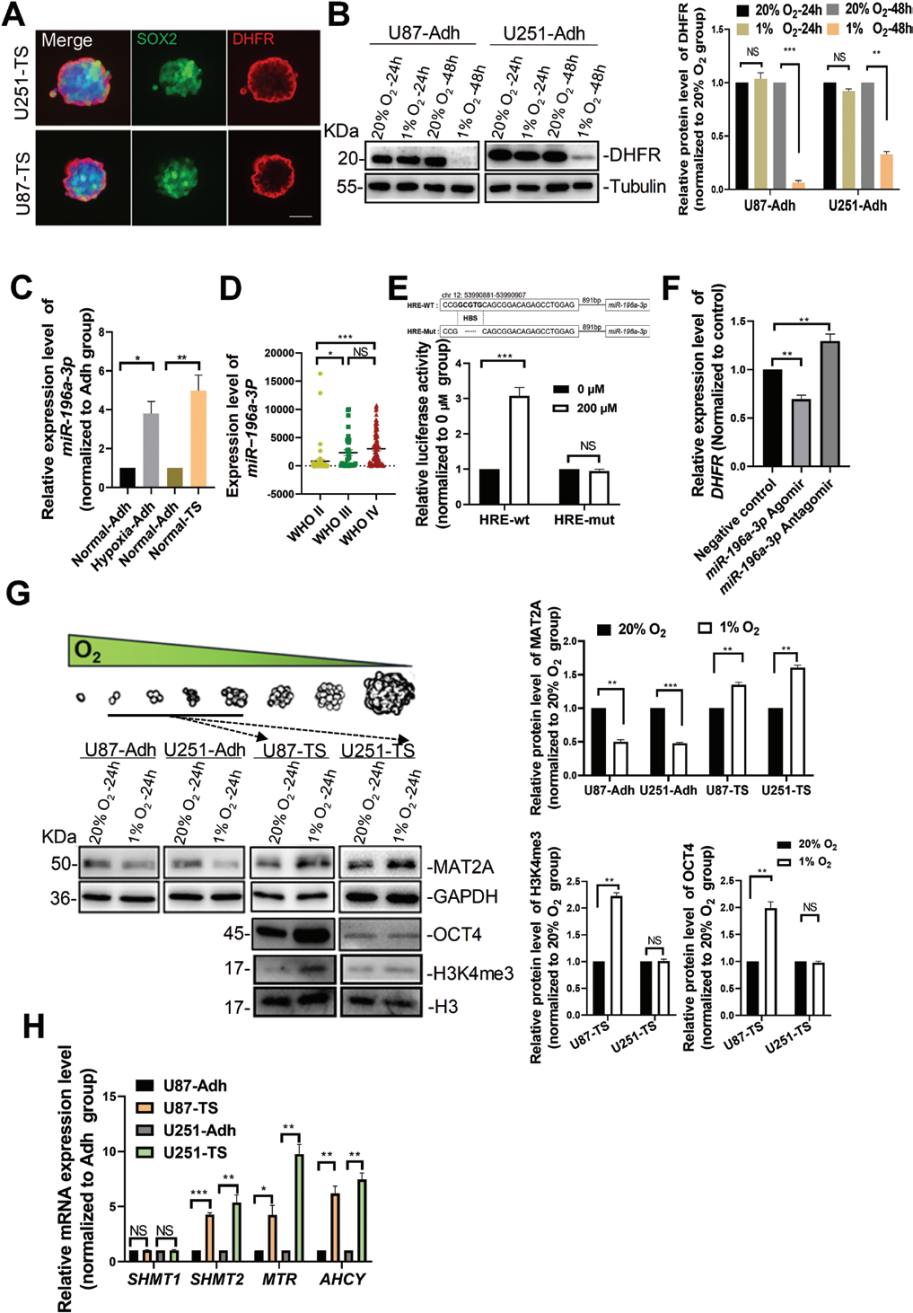
**Hypoxia is a key factor in altering the pattern of one-carbon metabolism.** (A) Representative images of DHFR (Red) and SOX2 (green) immunostaining of tumorspheres that were derived from U251-MG (top) and U87-MG (bottom) cell lines for 10 days. Scale bar, 100 μm. (B) Representative images and quantification of Western blots showing protein levels of DHFR in adherent tumor cells cultured under normal and hypoxic conditions for 24 or 48 h. Tubulin was used as a loading control. (C) Relative *miR-196a-3p* expression levels in adherent tumor cells from U87-MG cell line culture for 2 days under normal or hypoxic conditions. Tumorspheres from U87-MG cell line were cultured under normal conditions. *n* = 3 biologically independent experiments. (D) Expression levels of *miR-196a-3p* in glioma samples from CGGA database. WHO grade II glioma (*n* = 57), WHO grade III glioma (*n* = 32), WHO grade IV glioma (*n* = 90). (E) The schematic diagram showing a hypoxia-response element (HRE) upstream of the promoter of *miR-196a-3p*. HBS: HIF binding site. Lower panel, relative luciferase activities in HEK293T cells that were transfected with HRE-wt or HRE-mut luciferase plasmids, and then treated with 200 μM CoCl_2_ for 24 h. (F) Relative *DHFR* mRNA expression levels in adherent tumor cells from U87-MG cells which were transfected with *miR-196a-3p* agomir or *miR-196a-3p* antagomir and cultured for 2 days. (G) Protein levels of MAT2A, OCT4, and H3K4me3 in adherent tumor cells and tumorspheres cultured for 24 h under normal or hypoxic conditions. GAPDH or H3 was used as a loading control. (H) Relative mRNA expression levels of genes related to one-carbon metabolism in adherent tumor cells and tumorspheres by real-time reverse transcriptase-polymerase chain reaction (qRT-PCR). *n* = 3 biologically independent experiments. Data are presented as mean ± SEM. *P < 0.05, **P < 0.01, ***P < 0.001, NS: no significance.

Tumor spheroids have proliferating cells on their outer layer, quiescent cells on their inner layer, and necrotic cells in their core [25, 26]. In contrary to cells on the outer layer with more accessible access to oxygen and nutrients, cells on the inner layer have trouble in getting oxygen and nutrients and in exposing themselves to a higher concentration of carbon dioxide and other wastes [27, 28]. To investigate the mechanism of altered pattern of one-carbon metabolism of tumorsphere cells, we firstly analyzed the expression pattern of DHFR in tumorspheres. Interestingly, most cells on the outer layer of tumorspheres highly expressed DHFR, while cells in the inner layer barely expressed DHFR (Fig. 3A). We speculated that oxygen concentration may be a key factor in the regulation of DHFR expression pattern. To test this hypothesis, we compared the expression levels of DHFR in adherent tumor cells under hypoxia (1% oxygen concentration) versus normal oxygen concentration (20%). As expected, DHFR was dramatically down-regulated under hypoxia for 48 h (Fig. 3B), indicating that hypoxia represses the expression of DHFR.

Gene expression in response to hypoxia is commonly regulated by the HIFs. HIF-1α and HIF-2α are highly homologous and bind to similar hypoxia responsive elements (HRE) sequences (R/CGTG) in the promoters of hypoxia-regulated genes. Since HIFs are oxygen-dependent transcriptional activators, we reasoned that there may be an intermediate molecule bridging between HIF and DHFR, which not only directly inhibits the expression of DHFR but also is directly regulated by HIFs. MicroRNAs (miRNAs) are small non-coding RNAs that mediate gene silencing, and we predicted that several miRNAs (*miR-196a-3p*, *miR-329-5p*, *miR-381-3p*, and *miR-493-3p*) have HRE sequences in their promoter regions as well as binding sites on the 3ʹ UTR of *DHFR*. By analyzing the expression of miRNAs, we found that *miR-196a-3p* was significantly up-regulated in adherent cells under hypoxic conditions and in tumorspheres (Fig. 3C). In the CGGA database, the expression of *miR-196a-3p* is much higher in WHO grade II/III/IV glioma than that in healthy brain (Fig. 3D). These data indicate that the expression of *miR-196a-3p* is in response to hypoxia.

To test whether *miR-196a-3p* is regulated by HIF, we transfected HEK293T cells with the luciferase vector (pGL3-basic) containing the wild-type or mutated HRE of *miR-196a-3p* promoter, stimulated HEK293T cells with hypoxia, and measured the luciferase activity of both constructs. The luciferase activity was significantly increased in HEK293T cells transfected with wild-type HRE construct after hypoxia stimulation, compared to that in cells transfected with the mutant HRE construct (Fig. 3E). Thus, HIF induces *miR-196a-3p* transcription by binding to putative HRE sequence in the *miR-196a-3p* promoter. Next, we transfected U87-MG adherent cells with the *miR-196a-3p* agomir or antagomir, and confirmed that the expression of *DHFR* was down- and up-regulated by *miR-196a-3p* agomir and antagomir, respectively (Fig. 3F). Since the expression of *mir-196a-3p* was significantly upregulated in tumorspheres compared to adherent tumor cells (Fig. 3C), and the expression level of DHFR was significantly decreased in inner cells compared to that in outer cells of tumorspheres (Fig. 3A), we speculate that the HIF-*miR-196a-3p*-DHFR axis may play an important role in altering the pattern of one-carbon metabolism in tumorsphere cells under hypoxia conditions.

Since hypoxia supports the maintenance of GSCs, and GSCs have been found to exhibit a remarkable dependency on exogenous methionine [22], we then explored whether adherent tumor cells under hypoxia had a similar enhanced MAT2A expression as in GSCs. Surprisingly, the results showed that MAT2A expression was down-regulated in adherent tumor cells under hypoxia, indicating that the enhanced expression of MAT2A by hypoxia might be present only in GSCs. Next, we administered the stimulus of external hypoxia at the early stage (0–24 h) of tumorsphere formation to determine any changes in MAT2A expression. As expected, MAT2A was up-regulated in tumorspheres from both U87-MG and U251-MG cell lines that were stimulated by hypoxia, but the upregulation of stemness marker OCT4 and H3K4me3 was only observed in hypoxia-treated tumorspheres from U87 cell line (Fig. 3G), suggesting the importance of MAT2A in tumorsphere formation under hypoxia and that there may be an intrinsic difference in regulatory mechanism between these two GBM cell lines. During the formation of tumorspheres, the oxygen level is gradually decreased, which in turn induces the up-regulation of HIF [29]. We speculated that hypoxia is the key to causing the altered pattern of folate and methionine metabolism. Indeed, we found that the expression of metabolic enzymes *MTR*, *AHCY*, and *SHMT2* was elevated in tumorspheres compared to adherent tumor cells (Fig. 3H), which supports the idea that hypoxia is a key factor in altering the pattern of one-carbon metabolism in tumorsphere cells.

### Short-term methionine starvation impairs tumorigenicity of GSCs

**Figure 4.**
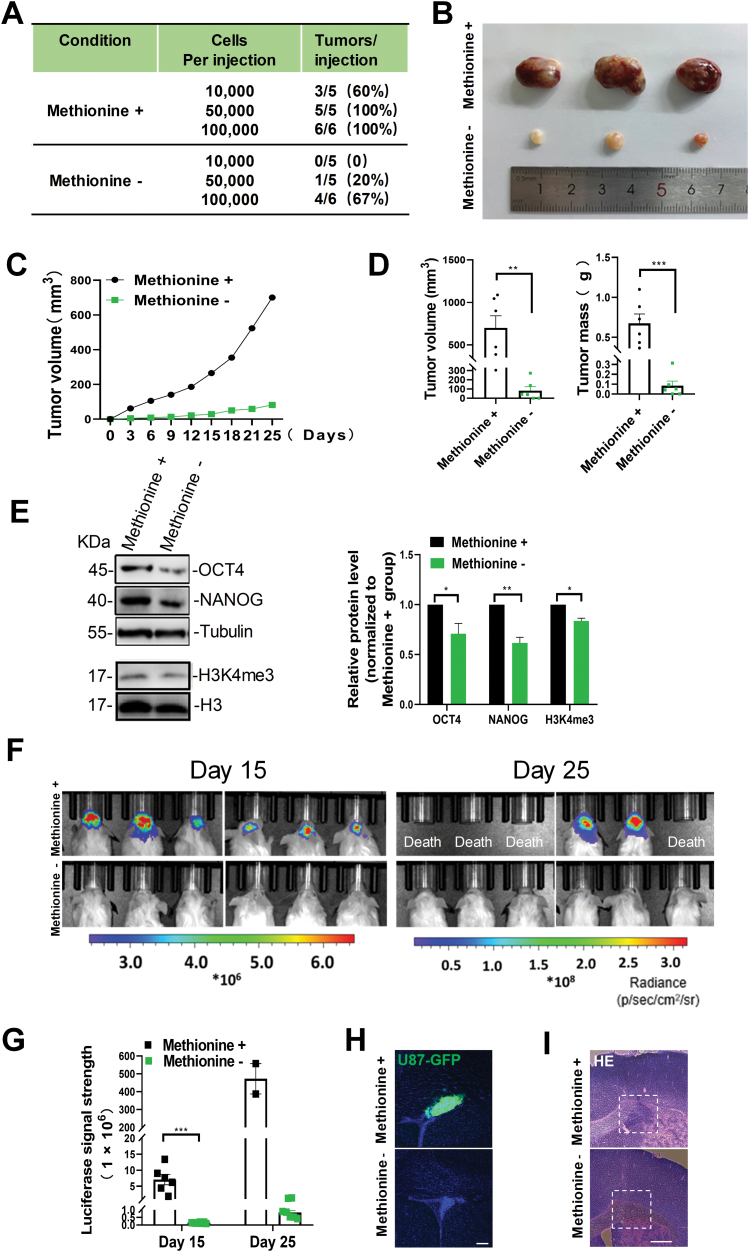
**Short-term methionine deprivation impairs GSCs tumorigenicity.** (A) Tumor formation rates of tumor-sphere-derived single cells transplanted subcutaneously into NSG mice. Tumorshperes were cultured in medium with or without methionine for 2 days. (B) Representative images of tumors that were formed by subcutaneously transplanted 100,000 single cells derived from tumorspheres which were cultured in medium with or without methionine. (C) Tumor growth curve for the transplantation of 100,000 single cells derived from tumor-spheres which were grown in medium with the complete formulation or methionine deprivation. (D) Quantifications of tumor mass and tumor volume at day 25 following subcutaneous implantation of 100,000 tumor-sphere-derived single cells. *n* = 6 biologically independent experiments. (E) Protein levels of OCT4, NANOG and H3K4me3 in tumors that were formed by transplanted tumor-sphere-derived single cells. Tubulin or H3 was used as a loading control. (F, G) *In vivo* bioluminescent images (F) and the quantification (G) of orthotopic xenografts derived from U87-GSCs which were cultured with or without methionine at the indicated time points. (H) Representative images showing the regions with implanted U87-GSCs (GFP^+^) that were cultured in medium with or without methionine. (I) Representative images of hematoxylin/eosin (HE) staining on coronal sections with orthotopic xenografts derived from U87-GSCs that were cultured in medium with or without methionine. The dotted white boxes indicate the U87-GSCs-implanted regions. White bars, 100 μm. Data are presented as mean ± SEM. *P < 0.05, **P < 0.01.

Because long-term (>7 days) deprivation of methionine leads to the general lethality of cells, we performed a transient 48-h starvation protocol to assess the prominence of methionine cycle metabolites in tumorsphere cells [22]. We subcutaneously xenografted tumorsphere cells with methionine starvation for 48 h into the immune-compromised mice (NSG) and found that these cells lost their tumor-forming ability, as evidenced by declined tumor formation rates (Fig. 4A) as well as reduced tumor sizes (Fig. 4B). Consistently, short-term methionine starvation significantly inhibited the growth rate of tumorsphere cells (Fig. 4C), which lead to a dramatic decrease in tumor mass (~8.4-fold) and in tumor volume (8.6-fold) at day 25 following subcutaneous implantation of 100,000 tumorsphere-derived single cells (Fig. 4D). Meanwhile, the protein levels of OCT4, NANOG, and H3K4me3 were significantly decreased in tumors that were formed by methionine-starvation-treated tumorsphere cells (Fig. 4E).

Next, we further tested the effect of methionine short-term starvation using an orthotopic tumor implantation model, in which U87-GSCs stably expressing both GFP and a firefly luciferase reporter were transplanted the right frontal lobe of NSG mice and tumors were measured by bioluminescent imaging using *In Vivo* Imaging System (Perkin-Elmer). Again, we observed a marked reduction in the growth of methionine-starvation-treated U87-GSCs orthotopic xenografts, as evidenced by a nearly 50-fold decrease in luciferase brightness at day 15, and a 600-fold decline in luciferase signal at day 25 after xenograft transplantation (Fig. 4F and G). Furthermore, GFP signal was almost undetectable in the brain with the transplantation of methionine-starvation-treated tumorsphere cells, but not in the brain with the transplantation of U87-GSCs that were cultured in medium with the complete formulation (Fig. 4H). Hematoxylin/eosin staining showed that methionine starvation correlated with dramatically decreased tumor size (Fig. 4I). Taken together, these results support that short-term methionine starvation impairs tumorigenicity of GSCs.

### FIDAS-5 impacts the tumorigenicity of GSCs

**Figure 5.**
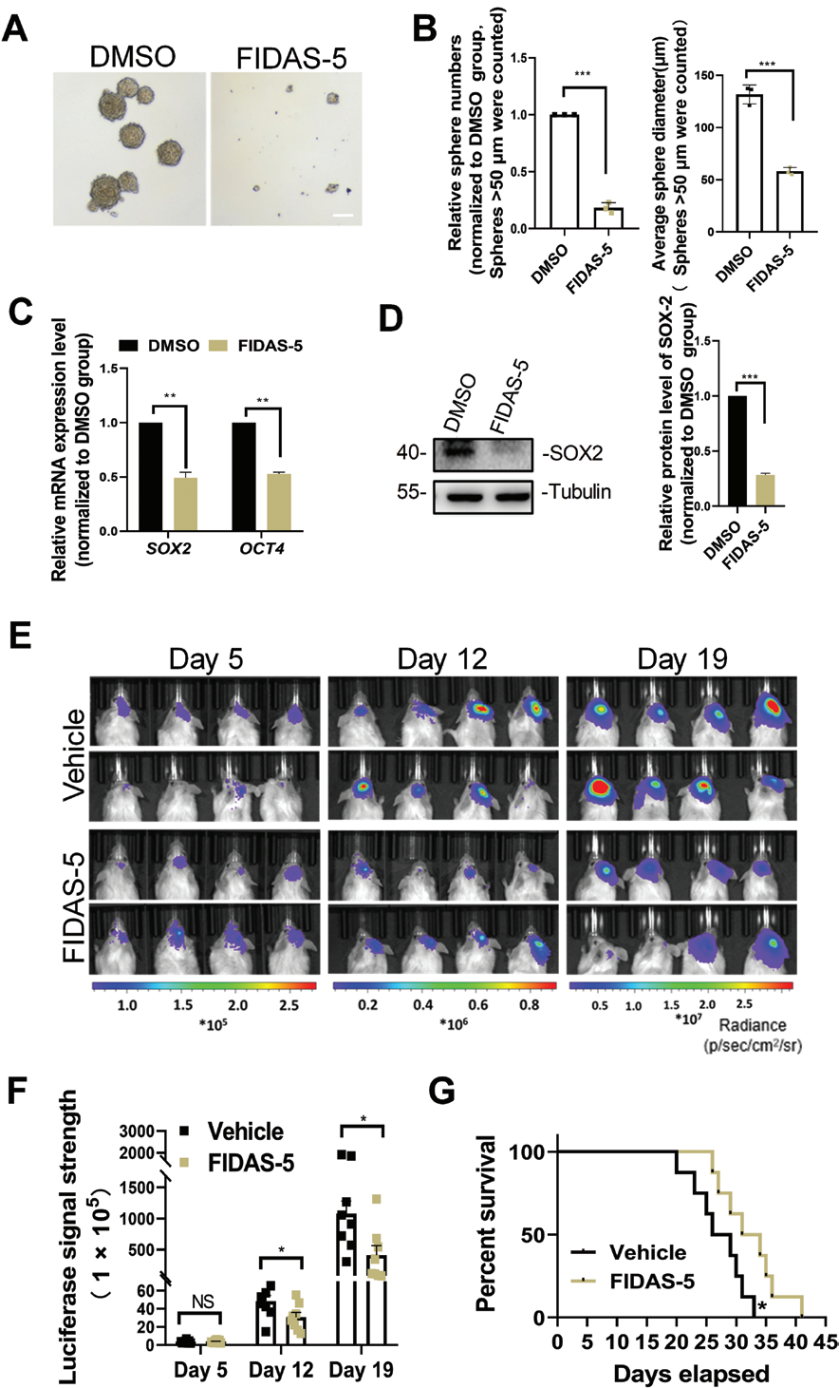
**FIDAS-5 suppresses GSCs tumorigenicity.** (A) Representative images of tumor-sphere formation by U251-MG cells that were exposed to 10 μM FIDAS-5 or DMSO for 10 days. White bar, 100 μm. (B) Quantifications of the numbers and diameters of tumorspheres that were cultured with 10 μM FIDAS-5. *n* = 3 biologically independent experiments. (C) qPCR analysis showing the relative mRNA levels of *SOX2* and *OCT4* in the tumorspheres that were treated with DMSO, FIDAS-5 or methionine deprivation for 10 days. GAPDH was used as an internal control. (D) Protein levels of SOX2 in tumorspheres exposed to 10 μM FIDAS-5 or DMSO for 10 days. Tubulin was used as a loading control. (E, F) *In vivo* bioluminescent images (E) and the quantification (F) of orthotopic xenografts derived from U87-GSCs treated with 40 mg/kg FIDAS-5 at the indicated time points. *n* = 8 biologically independent experiments. (G) Kaplan–Meier survival analysis of mice bearing GSC-derived xenografts of U87-GSCs that were treated with FIDAS-5. Data are presented as mean ± SEM. **P < 0.01, ***P < 0.001.

It has been reported that the MAT2A inhibitor FIDAS-5 perturbs methionine cycle activity and cellular methylation levels and induces a complete ablation of all histone methylation marks in tumorsphere cells [22]. Decreased H3K4 methylation locks neural stem cells in a pro-differentiation state [30] and global inhibition of histone methylation diminishes the tumorigenicity of lung tumor-initiating cells [22]. To further evaluate the methionine cycle as a therapeutic target of GSCs, lastly we tested whether FIDAS-5 impacts the tumorigenicity of GSCs. After the treatment of 10 μM FIADS-5 for 10 days *in vitro*, U87-MG cells showed a significant decrease in tumorsphere formation (Fig. 5A and 5B), together with reduced levels of *SOX2* and *OCT4* expression (Fig. 5C and 5D). These results indicate that MAT2A inhibition has a potential application for the prevention of GSCs tumorigenicity.

Next, we sought to test whether FIDAS-5 could impact the tumorigenic potential of GSCs *in vivo*. GSCs expressing luciferase were transplanted into the right frontal lobe of NSG mice through intracranial injection to establish orthotopic xenografts. We then treated the orthotopic xenografts daily with FIDAS-5 (40 mg per kg) or vehicle control, and observed that intraperitoneal injection of FIDAS-5 markedly inhibited the growth of xenografts (Fig. 5E and 5F) and conferred a significant survival benefit relative to the vehicle control (Fig. 5G), thus support that MAT2A inhibitor effectively suppresses GBM growth and enhances animal survival.

## Discussion

Gliobalstoma is a heterogeneous disease with different tumor cell populations as well as a complex microenvironment [31]. Alteration in cellular metabolism is a well-known hallmark of glioblastoma, which is driven by mutations in the genes such as the receptor tyrosine kinase (RTK) and isocitrate dehydrogenase (IDH) pathways [32, 33]. However, a comprehensive understanding of the interactions between upstream regulating molecules and metabolic reprogramming are lacking [34]. This study supports the idea that hypoxia is a key upstream regulator in altering the pattern of one-carbon metabolism in GBM tumorsphere cells.

Although antifolates have been used to treat a series of blood cancers in clinic [35], the association between folate and cancer risk remains controversial, as studies have demonstrated positive, negative, and neutral impacts of folate on cancer risk [36]. Exogenous methionine is required for GBM tumorsphere formation but not for the proliferation of monolayer cells, and cytosolic folate cycle genes (*DHFR*, *SHMT1*, and *MTHFD1*) are downregulated in U251-MG tumorspheres vs. monolayer cells [37]. Similarly, we found that DHFR is significantly down-regulated in tumorspheres derived from glioblastoma cell lines compared with adherent tumor cells. Additionally, folate-deprivation or inhibition of DHFR does not impact the formation and growth of GBM tumorspheres. In contrary, monolayer adherent cells derived from GBM cell lines cannot grow under DHFR inhibitor treatment. It is worthy to note that DHFR inhibition, either by Methotrexate (MTX) or EphB activation with synthetic ligands, has been previously reported to reduce the self-renewing potential of brain tumor-initiating cells [38]. Thus, in the context of tumor heterogeneity, the necessity of folate in GBM tumor growth may be cell-type-dependent and influenced by cellular microenvironments.

Hypoxia is a well-recognized tumor microenvironmental condition that is linked to poor patient outcome and resistance to therapies [39–44]. The tumorsphere is a solid, spherical formation with a gradual decrease in oxygen content from its outside to inside. Hypoxia leads to activation of the transcription factors HIF-1, which regulates gene expression within solid tumor cells, influencing tumor progression and treatment response [45, 46]. There is a growing body of evidence suggesting that hypoxia plays a central role in the prognosis of GBM patients [47]. As the main factor activated by hypoxia, HIF-1 activates the expression of many genes that are responsible for angiogenesis, cell invasion, autophagy, and metabolism [48, 49]. Our study provides evidence that *miR-196a-3p* is significantly up-regulated in tumorspheres and in adherent cells under hypoxic conditions, and HIF induces *miR-196a-3p* transcription by binding to HRE sequence in the *miR-196a-3p* promoter. Consistently, Takkar and colleagues also reported that hypoxia-induced upregulation of *miR-196a-5p* is also existed in glioma cell lines [50]. Since *miR-196a-3p* and *miR-196a-5p* are generated from the 5p and 3p arm of the same pre-miRNA, respectively, we speculate that these two miRNAs share the same upstream regulator, HIF-1. Although the present study clearly demonstrates that *miR-196a-3p* agomir inhibits and *miR-196a-3p* antagomir increases the expression of DHFR in adherent cells, future investigations are required to see whether GBM tumorspheres with DHFR re-expression from *miR-196a-3p* antagomir no longer rely on exogenous methionine. Importantly, *miR-196a* has been reported to be upregulated in various cancers [51], it would therefore be interesting to examine whether the HIF-1-*miR-196a*-DHFR regulatory axis also exists in other kinds of cancers.

It was reported that the mitochondrial isoform of SHMT in one-carbon metabolism, SHMT2, is upregulated in Myc-overexpressing neuroblastoma and GBM cell lines, but not other cancer cell lines (SH-SY5Y, HEK293T, H1299, and HT1080) upon hypoxia [52]. SHMT2 maintains the cellular NADPH/NADP^+^ ratio when cells are subjected to hypoxia, and depletion of SHMT2 leads to cell death by increasing ROS levels in hypoxic cells [52]. Interestingly, a few other cancer types, such as some human breast cancers, adrenocortical carcinoma and kidney chromophobe cell carcinoma, also exhibit an increased SHMT2 expression in metastatic tumor tissue during cancer progression [53]. Our data also revealed that hypoxia induces up-regulation of SHMT2 and MAT2A in tumorsphere cells, but not in monolayer adherent cells derived from GBM cell lines. Short-term starvation for methionine or exposure to the MAT2A inhibitor FIDAS-5 leads to dramatic impairment of the tumorigenic potential of GBM tumorsphere cells. Similar observations have been made in lung tumorsphere cells, and small-molecule perturbation of the methionine cycle diminishes the tumorigenicity of tumor-initiating cells [22]. High expression levels of MAT2A are also observed in colorectal cancer, leukemia, lymphoma, nasopharyngeal carcinoma, melanoma, ovarian carcinoma, prostate adenocarcinoma and breast cancer than in their corresponding normal tissues [22]. Therefore, limiting methionine intake or targeting MAT2A may be a promising strategy for treating GBM and other cancer types. Despite further investigation into possible side effects of limiting methionine intake and/or targeting MAT2A is merited, there is strong evidence showing dietary methionine restriction may not only produce lifespan extension but also prevent several chronic diseases and cancer [54, 55]. Intracellular methionine may mediate the effects of a variety of nutrients and genetic interference on the life span of the body, and glucose methionine crosstalk may serve as a special mechanism to coordinate the nutritional status of the body and cell translation/growth [56]. However, it is worth noting that GBM displays a complex cellular heterogeneity containing GSCs and non-GSCs [2]. In the present study, our results suggest that GSCs are more dependent on the methionine cycle, while monolayer adherent cells mainly rely on the folate cycle. Thus, the integrative therapeutic strategy of targeting both folate and methionine cycles at the same time is likely to be a promising approach for treating GBM.

**Figure 6.**
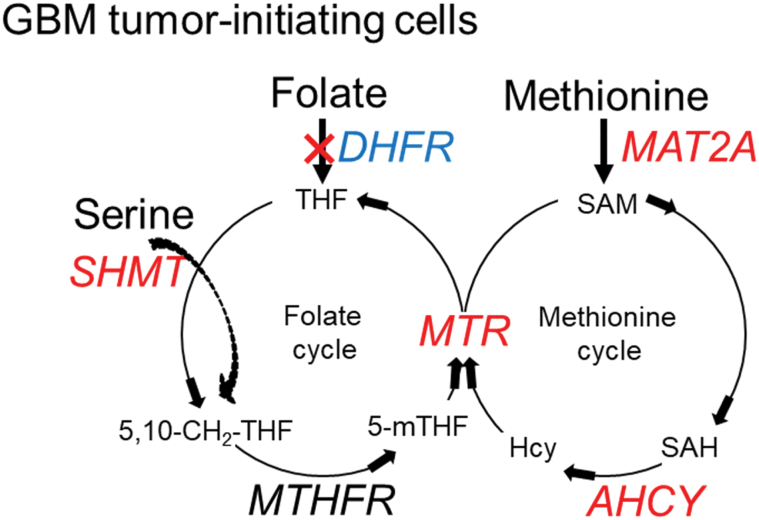
**Graphical summary.** The schematic diagram demonstrating the regulatory loops among genes involved in one- carbon metabolism in GSCs. Genes with upregulation and downregulation are in red and blue, respectively. 5,10-CH_2_-THF: 5,10-methylenetetrahydrofolate.

In conclusion, our data support the idea that GBM tumor-initiating cells have a decreased demand for folate but an elevated dependency on methionine cycle activity under hypoxic conditions (Fig. 6). Our findings suggest that methionine cycle inhibition via limiting methionine intake or targeting MAT2A reduces the tumorigenicity of GBM tumor-initiating cells, highlighting the great therapeutic potential of drugs against methionine cycle activity for the eradication of GSC-like cells in patients with GBM.

## Research limitations

Although the present study provides evidence that GBM tumor-initiating cells have an elevated dependency on methionine cycle activity under hypoxic conditions, however, further investigations are needed to validate the HIF-*miR-196a-3p*-DHFR axis in regulating one-carbon metabolism in tumorspheres and *in vivo* as well. To better understand the molecular mechanisms of GBM tumorigenicity, single-cell and spatial multi-omics are indispensable to provide a comprehensive picture of the altered gene regulatory network in future research. Finally, the effects of long-term methionine deficiency on cell death and proliferation of both tumorspheres and adherent tumor cells are worthy to be explored in the future before translating findings from cell lines/animals to human patients with GBM.

## Methods

### Cell culture

All glioma cells (U87-MG, U251-MG, A712) used in this study were obtained from ATCC and cultured in Dulbecco’s Modified Eagle medium (Gibco, USA) containing 10% fetal bovine serum (HyClone, USA). U87-MG were transduced with lentivirus expressing luciferase and GFP, and followed by fluorescence-activated cell sorting (FACS). The medium does not contain folate or methionine were customized (Zomanbio, #ZY105/ZY106). All cell lines used in this study were analyzed by short tandem repeat typing and mycoplasma serology, passaged 2–6 times for experimental use, and revived every 3 months.

### Sphere formation assay

Sphere formation assay was performed as previously described [11, 15, 57]. GSCs were cultured in Low Attachment Multiple Well Plates (Corning, #3471) at a density of 1000 cells/well. U87-MG, U251-MG, and A172 were cultured in neurobasal medium containing B27, basic fibroblast growth factor (10 ng/ml), and epidermal growth factor (10 ng/ml). After 10 days of culture, the tumorspheres were measured and analyzed.

### Cell metabolic analysis

5-mTHF (Novoprotein, #DG94099Q), Hcy (ABclonal, #RK09092), SAM (MEIMIAN, #MM-13267H1), and SAH (MEIMIAN, #MM-13268H1) were detected according to the manufacturer’s instruction. Briefly, cell culture media were collected using a pipette, followed by centrifugation at 4°C for 20 min at 1500 rpm. The clear supernatant was then collected for metabolic assay immediately. The concentration was calculated with the standard curve method.

### Plasmid construction and luciferase assays

The promoter contains the HRE sequence of *miR-196a-3p* was cloned into the luciferase expression plasmid (pGL3-basic). Primers used for cloning promoter of *miR-196a-3p* were as follows: HRE-wt (forward 5ʹ-CGGATCGATAGAGCACAGGCAGAATCGTT-3ʹ, reverse 5ʹ-CGCGCTAGCCGGTTTAGGCTTCTCAGCGA-3ʹ), HRE-mut (forward 5ʹ-TGTGTGTGTTTTGTGTTTCTCCTAGAGCCTTTCTAGAACGTC-3ʹ, reverse 5ʹ-AGAAAGGCTCTAGGAGAAACACAAAACACACACATCCA CAC-3ʹ). All plasmid constructs were then verified by Sanger sequencing. Luciferase assays were performed as previously described [58, 59]. In brief, HEK293T cells in 48-well plates were transfected with HRE-wt or HRE-mut plasmid using Lipofectamine 2000 (Invitrogen, Carlsbad, CA, USA), and were then treated with 200 μM CoCl_2_ for 24 h. All Luciferase readings were recorded using the Luciferase Reporter 1000 System (Promega, Madison, WI, USA) following the manufacturer’s instructions.

### Hypoxia induction

In order to induce hypoxia, cells were cultured in hypoxia chambers with 1% O_2_, 5% CO_2_, and 94% N_2_.

### Immunofluorescence staining

Immunofluorescence staining was performed as described previously [60]. Briefly, cells were fixed with 2% PFA for 20 min at room temperature, followed by phosphate-buffered saline (PBS) washing. Samples were then blocked with 2% bovine serum albumin (BSA) for 1 h after permeabilization with 0.5% Triton-X in PBS for 20 min at room temperature. After incubating with primary antibodies overnight at 4°C, samples were washed with PBS, followed by incubation with appropriate fluorescent-labeled secondary antibodies for 2 h at room temperature. DAPI was used to stain nuclei. Confocal images were obtained on a ZEISS 710 confocal laser-scanning microscope. Image analyses and quantification were performed using ImageJ software V1.53 (NIH, Bethesda, MD, USA).

### Western blotting

Western blotting was performed as described previously [60]. Cells or tissues were lysed with RIPA buffer (P0013B; Beyotime, Shanghai, China). Protein samples were separated on 8%–12% SDS-PAGE gels and transferred to polyvinylidenefluoride (PVDF) membranes (Millipore, Hongkong, China). The PVDF membranes were then blocked in Tris-buffered saline-Tween (TBS-T) containing 3% milk and incubated with primary antibodies MAT2A (Abclonal, #A19272; 1:1000), DHFR (Abclonal, #A1607; 1:1000), SOX2 (Santa Cruz, #sc-17320; 1:500), OCT4 (Abclonal, #A7920; 1:1000), or NANOG (Abclonal, #A3232; 1:1000) at 4°C overnight. PVDF membranes were then incubated with horseradish peroxidase-conjugated secondary antibodies (1:5000) at room temperature for 2 h. Finally, the immunoreactive proteins were treated with enhanced chemiluminescence reagent (Pierce ECL; Thermo Fisher, Shanghai, China). The 5200 Tanon™ Chemi-Image System was used to obtain the images of the blots, and the band intensity of the blots was analyzed using the software ImageJ.

### Tumor implantation and collection

Tumor implantation was performed as previously described [22]. 1 × 10^5^ single U87-GSCs were mixed in a 1:1 mixture of serum-free DMEM/F12 and Matrigel (BD, #354234) and injected subcutaneously into the flanks of 4- to 6-week-old NSG mice (Jackson Laboratories). About 4 weeks later, or when tumor sizes exceeded 2 cm in diameter, mice were killed and tumors were collected for analysis. All animal procedures followed the ethical guidelines for the care and use of experimental animals, and all experiments were approved by the Animal Committee of the Institute of Zoology, Chinese Academy of Sciences.

### Tumor measurements

Tumor volume was calculated by the formula 0.5 × *l* × *w*^2^, where *l* and *w* are tumor length and width, respectively. The formed tumors were also analyzed by hematoxylin and eosin staining as described previously [12].

### Establishment of GSC-derived intracranial GBM xenografts and FIDAS-5 treatment

Orthotopic GBM xenografts were established through intracranial transplantation of GSCs as described previously [61–63]. Total of 5000 single U87-GSCs expressing luciferase and GFP were injected into the right frontal lobe of mice. Mice were undergone one of the following treatments: vehicle control (i.p.), FIDAS-5 (40 mg/kg, i.p., TargetMol, #T11285) starting on the fifth day after tumor implantation. Xenograft growth was monitored by bioluminescent imaging using *In Vivo* Imaging System (Perkin-Elmer). Mice were sacrificed at the indicated time points or upon manifestation of neurological symptoms.

### qRT-PCR

Total RNA was isolated with TRIzol reagent (Invitrogen, Carlsbad, CA, USA) according to the manufacturer’s instructions. RNA quality was determined with the Thermo Nano Drop 2000 spectrophotometer to assess 260/280 and 260/230 nm ratios. All RNA samples met a 260/280 ratio > 2.0 and 260/230 ratios in the range of 2.0–2.2. cDNA was generated from reverse transcription of 2 µg total RNA using a Transcriptor First Strand cDNA Synthesis Kit (TransGen Biotech, Beijing, China). cDNA was quantified using the SYBR Green assay, and the relative gene expression levels were calculated against GAPDH or U6 by using the ∆∆Ct method. Primers for qRT-PCR were as follows: MTR (forward 5ʹ-AGCAGTCTACAGGCATTAGG-3ʹ, reverse 5ʹ-AATTGGAGAAGTAGAGGCCTG-3ʹ), AHCY (forward 5ʹ-GCATCTCT GAGGAGACCACG-3ʹ, reverse 5ʹ-TTGCTCTTGGTGACGGAGTC-3ʹ), SHMT1 (forward 5ʹ-CAAAGACAGTGATGTTGAGGT-3ʹ, reverse 5ʹ-CCATAGTATCTCTGGCCCG-3ʹ), SHMT2 (forward 5ʹ-CTGAGATGT GGGAGTTGCT-3ʹ, reverse 5ʹ-TGCAGAAGTTCTCTGAGGC-3ʹ), *miR-196a-3p* (forward 5ʹ-CGGCAACAAGAAACTGCCTG-3ʹ, reverse 5ʹ-CCGGGAGGTTTTCTGGGTTTC-3ʹ).

### Apoptosis assay

Apoptosis assay was performed as described previously [60]. Terminal deoxynucleotidyl transferase dUTP nick end labeling (TUNEL) staining (Beyotime Biotechnology, Shanghai, China) was performed to detect the apoptosis. Briefly, DNase I treated was used as a positive control, 2 μl DNase I was added to the medium and placed at 37°C for 10 min. Cells were fixed in 2% PFA and then washed with PBS for 10 min, incubated in 2% BSA and 0.25% triton X-100 for 30 min at room temperature. Cells were then incubated with 200 µl TUNEL reaction mixture at 37°C for 1 h, Finally, cells were washed with PBS three times for 10 min.

### CCK-8 assay

CCK-8 assay was performed according to the manufacturer’s instruction (Beyotime Biotechnology, Shanghai, China). Briefly, 10 μl of CCK-8 solution was added into each well, and cells were then incubated at 37°C and 5% CO_2_ for 2 h. Finally, the absorbance at 450 nm was measured using a microplate reader.

### CGGA database

All gene expression data about glioma patients were downloaded from the Chinese Glioma Genome Atlas (CGGA) database (www.cgga.org.cn).

### Research ethics

This study was approved by the Ethics Committee of the Institute of Zoology, Chinese Academy of Sciences and with the written consent of all participants. All experiments involving animals were performed following the animal protocol approved by the Animal Committee of the Institute of Zoology, Chinese Academy of Sciences.

### Data availability

The datasets used and/or analyzed during the current study are available from the corresponding author on reasonable request.

### Statistical analysis

All statistical data analyses were performed using the software GraphPad Prism v7.2 (GraphPad, San Diego, CA, USA). Datasets were analyzed for significance using a one-way ANOVA with Dunnett’s multiple comparisons test. All data are presented as mean ± SEM. When a *P*-value is less than 0.05, the results are determined as statistically significant.
